# P-1068. Inhibitory Effect of EDTA on Metallo β-Lactamase Producing Gram-Negative Pathogens Recovered from Cancer Patients with Bloodstream Infections

**DOI:** 10.1093/ofid/ofae631.1256

**Published:** 2025-01-29

**Authors:** Issam I Raad, Joel Rosenblatt, Y Lan Truong, Bahgat Z Gerges, Ying Jiang

**Affiliations:** MD Anderson UT, Houston, Texas; MD Anderson UT, Houston, Texas; UT MD Anderson Cancer Center, Houston, Texas; MD Anderson UT, Houston, Texas; MD Anderson, UT, Houston, Texas

## Abstract

**Background:**

The spread of metallo β-lactamase (Mβ-L) producing Gram negative pathogens is an important worldwide medical problem. It presents the broadest-spectrum β-lactam resistance of all carbapenemase-producing organisms leaving few remaining treatment options. Previously, adding EDTA, a potent metallo-chelator, was shown to restore activity for antibiotic combinations against Mβ-L pathogens isolated from patients with urinary tract and bronchial infections. In this study, we evaluated *in vitro* efficacy of EDTA alone and in combination with different β-lactam/β-lactamase inhibitors against Mβ-L isolates recovered from cancer patients with bloodstream infections (BSIs).

**Figure:**
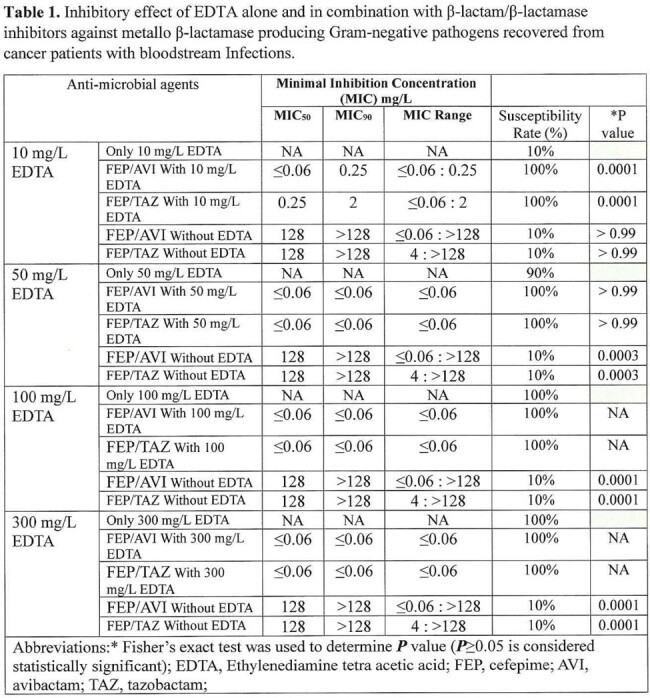

**Methods:**

: EDTA over its concentration range was tested alone and in combination with cefepime (FEP)/Avibactam (AVI), and FEP/Tazobactam (TAZ) against ten Mβ-L blood clinical isolates {*E.coli (n=6)*, *K. oxytoca (n=2)*, *P. aeruginosa* (n=1), and *E. cloacae* (n=1)}. CLSI approved broth microdilution methods were used to measure MIC. MIC_50_, MIC_90_, MIC ranges, and susceptibility rate calculations were made according to CLSI 2022. The provisional breakpoints susceptibilities of FEP/AVI, FEP/AVI/EDTA are ≤8/4 while the provisional breakpoints for FEP/TAZ, and FEP/TAZ /EDTA are =≤2/4. Statistically, significant differences were determined using Fisher’s exact test (***P***≤0.05 is considered significant).

**Results:**

**Table 1.** presents MIC_50_, MIC_90_, MIC ranges, susceptibility rates, and ***P*** values comparing EDTA with different concentrations alone and in combination with a range of FEP/AVI, and FEP/TAZ dilutions against ten Mβ-L bloodstream isolates.

**Conclusion:**

EDTA alone at 100 mg/L, and 300 mg/L concentrations inhibited 100% of Mβ-L blood isolates, while 10 mg/L, and 50 mg/L inhibited 10%, and 90% of tested isolates respectively. The combination of 10 mg/L EDTA with FEP/AVI, and FEP/TAZ, synergistically improved the susceptibility rates from only10% (without EDTA) to 100% (at only10 mg/L EDTA). The differences in MICs, and susceptibility rates between the triple and double combinations were highly significant (*P* < 0.0001). Further studies are needed to show the synergy against a larger number of Mβ-L Gram negative BSI pathogens as well as to clinically verify these benefits.

**Disclosures:**

**All Authors**: No reported disclosures

